# Challenges in functional hydrogel application for chronic tympanic membrane perforation: practical limitations and lessons learned

**DOI:** 10.1007/s13534-025-00519-y

**Published:** 2025-10-29

**Authors:** Gan-Erdene Narantsolmon, Yu-Jung Hwang, Min Ji Kim, Jin Young Hong, Soo Bin Yoon, Bayarmaa Enkhbat, Chang Hee Min, Myoung Ju Kim, Myung-Whan Suh, Young Bin Choy

**Affiliations:** 1 Department of Otorhinolaryngology, Seoul National University Hospital, Seoul, 03080 Republic of Korea; 2 Department of Otorhinolaryngology, First Central Hospital of Mongolia, Ulaanbaatar, 210648 Mongolia; 3 Department of Pathology and Forensic Medicine, Mongolian National University of Medical Science, Ulaanbaatar, 14210 Mongolia; 4 Interdisciplinary Program in Bioengineering, College of Engineering, Seoul National University, Seoul, 08826 Republic of Korea; 5 Integrated Major in Innovative Medical Science, Seoul National University, Seoul, 03080 Republic of Korea; 6 Institute of Medical and Biological Engineering, Medical Research Center, Seoul National University, Seoul, 03080 Republic of Korea; 7 Department of Biomedical Engineering, Seoul National University College of Medicine, Seoul, 03080 Republic of Korea; 8 Innovative Medical Technology Research Institute, Seoul National University Hospital, Seoul, 03122 Republic of Korea; 9 ToBIOS Inc., 3F, 9-7 Seongbuk-ro 5-gil, Seongbuk-gu, Seoul, 02880 Republic of Korea

**Keywords:** Tympanic membrane perforation, Hydrogel, Manganese porphyrin, In situ gelation, Transtympanic delivery, Biocompatibility

## Abstract

Chronic tympanic membrane (TM) perforations often persist due to oxidative stress and hypoxia in the middle ear. A minimally invasive, biocompatible hydrogel addressing these challenges could serve as an effective therapeutic dressing. A thiolated chitosan (CS-SH) and manganese porphyrin (MnP)-conjugated polyethylene glycol maleimide (MnP-PEG-MAL) hydrogel (Ch_MnP) was developed through in situ gelation via Michael addition reaction. Its mechanical properties and antioxidant activities (SOD- and CAT-like), were evaluated in vitro. For in vivo testing, Ch_MnP hydrogel was transtympanically injected into a chronic TM perforation rat model. Efficacy and safety were assessed using endoscopy, 3D computed tomography (CT), auditory brainstem response (ABR) testing, and histological analysis. The hydrogel exhibited optimal porosity and a swelling ratio of ~ 155%, making it well-suited as a wound healing scaffold. MnP incorporation enhanced reactive oxygen species (ROS) scavenging and O₂ generation under oxidative conditions. However, in vivo application showed no apparent improvement in TM regeneration, ABR thresholds, or histological outcomes. CT revealed a substantial hydrogel volume loss over 3 weeks, indicating significant water loss. This dehydration compromised the hydrogel’s structural integrity and functionality, diminishing its role as a scaffold and therapeutic agent. The Ch_MnP hydrogel exhibited excellent biocompatibility and antioxidant properties, with potential to alleviate chronic inflammation in TM perforation. However, healing of the perforation was not observed during the study period, primarily due to dehydration in the dry middle ear. These findings underscore the importance of maintaining a hydrated environment to enhance the therapeutic efficacy of hydrogel-based TM perforation treatments.

**Supplementary Information:**

The online version contains supplementary material available at 10.1007/s13534-025-00519-y.

## Introduction

Chronic otitis media (COM) is one of the most common ear disorders, affecting 39–200 million individuals worldwide [1]. Perforation of the tympanic membrane (TM) leads to recurrent bacterial invasion into the middle ear, which is usually semi-sterile in normal subjects. Thus, COM patients often experience chronic recurrent pus drainage from the ear and hearing loss, which significantly impacts their quality of life. In severe cases, COM can lead to life-threatening complications such as intracranial abscess, thrombophlebitis, and/or meningitis. The mortality rate from these intracranial complications can be as high as 35% [2].

Therefore, it is essential to reconstruct the TM by patching the perforation with muscle fascia to prevent recurrent bacterial invasion into the middle ear. This step, called tympanoplasty, is a crucial part of tympanomastoidectomy that largely determines the overall success of the surgical treatment. However, the initial graft acceptance rate can be as low as 65% [3]. The regenerative capacity of the TM tissue differs between chronic and acute disorders. The first seven days are critical for TM wound healing, determining whether a perforation will close or progress to a chronic state [4]. Acute perforations often heal spontaneously due to the TM’s inherent regenerative potential. The migration and proliferation of keratinocytes from progenitor cells located at the TM’s attachment to the malleus handle play a crucial role in this process [4]. In contrast, patients with COM experience a loss of regenerative and self-healing capacity in the TM. As a result, surgical intervention is often required, and incomplete tympanoplasty techniques may lead to recurrence.

Given these factors, a biocompatible hydrogel can serve as an effective wound dressing for the treatment of TM perforation. Since the TM is difficult to access, a hydrogel that can undergo gelation in situ immediately after injection at the site of the defect would offer the additional advantage of noninvasive delivery to the target area. To promote TM healing, effective removal of reactive oxygen species (ROS) and an adequate O_2_ supply can be also essential for optimizing the local microenvironment in chronic wounds of the perforated TM [5]. ROS may induce apoptosis in keratinocytes while interfering with native progenitor cells, contributing to chronic TM perforation. Furthermore, chronic TM perforations are usually associated with local microvascular deficiencies, which compromise blood supply and create a hypoxic microenvironment [6]. Reduced oxygen levels may also hinder the proliferation of progenitor cells necessary for TM regeneration. In this regard, a hydrogel made of functional materials can help regulate these factors, creating a favorable environment for TM regeneration.

Therefore, we assessed the applicability of in situ hydrogel made of functional materials with catalytic activity capable of converting ROS into O_2_ for the treatment of chronic TM perforation. The hydrogel herein (Ch_MnP hydrogel) could be gelled via a Michael Addition click reaction, in which thiolated chitosan (CS-SH) was used as the polymeric backbone and mixed with manganese porphyrin (MnP) modified with maleimide groups (MnP-PEG-MAL) as the cross-linker [7]. Chitosan has been widely used in hydrogel wound dressings owing to its excellent biocompatibility and cell-adhesive properties [8, 9]. Moreover, manganese porphyrin (MnP) provides a distinct advantage in wound healing due to its strong catalytic activity in ROS scavenging and oxygen generation, making it highly promising for wound regeneration [10, 11]. Since MnP is integrated into the chitosan hydrogel matrix, the resulting Ch_MnP hydrogel exhibits sustained ROS-scavenging and O_2_-generating effects.

We first evaluated the mechanical properties and catalytic efficacy of the hydrogel in vitro under chronic TM perforation-mimicking conditions. Then, we assessed its in vivo applicability using an animal model of chronic TM perforation [1]. Due to its in situ gelation property, the CS-SH and MnP-PEG-MAL, each in solution, could be injected into the bulla via a transtympanic route with almost no invasiveness, where they mix and subsequently gel to form the Ch_MnP hydrogel. We then evaluated the efficacy and safety of the Ch_MnP hydrogel in the bulla by assessing the endoscopic images, 3D computed tomography (CT) images, as well as hearing thresholds for click and 16 kHz tones. Finally, the histology of the middle and external ear tissues was evaluated after sacrificing the animals.

## Materials and methods

### Materials

Chitosan oligosaccharides (MW, 3500 Da) and 5,10,15,20-tetrakis(4-carboxyphenyl)-porphine-Mn(III) (Mn-TCPP; > 98%) were obtained from Kittolife (Pyeongtaek, Korea) and Por-Lab (Scharbeutz, Germany), respectively. NH_2_-PEG-maleimide (MW: 2000 g/mol, > 99%) and 4arm-PEG-maleimide (4arm-PEG-MAL; MW: 10,000 g/mol, > 95%) were purchased from JenKem Technology (TX, USA). N-Hydroxy succinimide (NHS; > 98%), 3,3-dithiodipropionic acid (99%), dialysis tubing (benzylated, 2 kDa molecular weight cutoff (MWCO)), O-(benzotriazol-1-yl)-N,N,N′,N′-tetramethyluronium hexafluorophosphate (HBTU; > 98%), dimethyl sulfoxide (DMSO; anhydrous; 99.9%), hydrogen peroxide (H_2_O_2_; 30% w/w in H_2_O) were purchased from Sigma-Aldrich (St. Louis, MO, USA). 1-(3-Dimethylaminopropyl)-3-ethylcarbodiimide (EDAC; > 98%) and N, N-diisopropylethylamine (DIPEA; > 99%) were purchased from TCI (Chuo-ku, Japan). Dithiothreitol (DTT; > 99%), snakeskin™ dialysis tubing (7 kDa MWCO), fetal bovine serum (FBS), and penicillin–streptomycin (10,000 U/ml) were obtained from Thermo Fisher Scientific (MA, USA). Disodium salt dihydrate (EDTA) was purchased from Santa Cruz Biotechnology (TX, USA). Mytomycin C was purchased from Thermo Fisher Scientific (MA, USA). A hematoxylin & eosin (H&E) staining kit was purchased from Abcam (Cambridge, UK). Zoletil50 was purchased from Virbac Korea (Seoul, South Korea).

### Synthesis and characterization of functional polymers.

The CS-SH and MnP-PEG-MAL were synthesized as described in our previous study, with modifications [7]. Briefly, to synthesize thiolated chitosan, 100 mg of chitosan oligosaccharide (CS), 60 mg of 3,3-dithiodipropionic acid, 64.4 mg of NHS and 107.3 mg of EDAC·HCl were dissolved in 10 ml of distilled water. The reaction was conducted at 60 °C and pH 5 for 12 h. Then, 400 mg of DTT was added, and the mixture was stirred for 3 h under dark conditions. The resulting solution was purified by dialysis in 5 mM HCl using a 2 kDa MWCO membrane for 5 days, and subsequently freeze-dried to yield the final CS-SH. To synthesize MnP-PEG-MAL, 27 mg of Mn-TCPP, 16.2 mg of NH_2_-PEG-MAL, 324 mg of HBTU, and 125 μl of DIPEA were dissolved in 9.6 ml of anhydrous DMSO and stirred at room temperature for 1 h. The resulting solution was dialyzed using 7 kDa MWCO tubing, sequentially in DI water for 24 h, 90% methanol for 3 h, and again in DI water for an additional 4 days. The purified solution was subsequently freeze-dried to obtain MnP-PEG-MAL in solid form.

The thiol content of the lyophilized CS-SH was quantified using Ellman’s assay according to standard protocols [12]. The Fourier transform infrared (FT-IR) spectra for both CS and CS-SH were obtained at room temperature over the range of 4000 − 600 cm^−1^ with a spectral resolution of 8 cm^−1^ (Tensor27, Bruker, Billerica, MA, USA). The FT-IR spectra of Mn-TCPP, NH_2_-PEG-MAL, and MnP-PEG-MAL were also obtained under the same conditions. Nuclear magnetic resonance (^1^H NMR) spectroscopy was performed at room temperature using DMSO-d6 as the solvent to characterize the chemical structure of MnP-PEG-MAL (Avance III HD 400, Bruker, Germany).

### Hydrogel preparation

The Ch_MnP hydrogel was prepared via in situ gelation by mixing the CS-SH solution with the solution containing maleimide-functionalized compounds [7]. Each solution was separately prepared in phosphate buffered saline (PBS; pH 7.4) at equimolar concentrations of thiol and maleimide groups (16.8 mM). The pH of the CS-SH solution was adjusted to 6.5. In the maleimide solution, the molar ratio of MnP-PEG-MAL and 4-arm PEG-MAL was set to 75:25 and 0:100 to produce the hydrogel with and without MnP (Ch_MnP(+) and Ch_MnP(-), respectively). Considering injection into the bulla inside the ear, each solution was loaded into a dual syringe and then injected through a 5 cm mini-volume line (1 mm inner diameter), where it formed a gel upon exiting a 23-gauge needle. To maintain sufficient fluidity under these conditions, a 12 wt% CS-SH solution combined with 3 v/v% 1 N NaOH was employed.

### Hydrogel characterization

The cross-sectional morphology of the hydrogels was analyzed by field-emission scanning electron microscopy (FE-SEM; SGMA, Carl Zeiss, Cambridge, UK). The hydrogel was lyophilized and sliced to expose its cross-section [13]. The swelling ratio of the hydrogel was assessed using a standard method [14]. A 60 μl of the hydrogel was prepared as described above, and then fully submerged in PBS (pH 7.4) at 37 °C for 48 h. The swelling ratio was calculated using the following equation:

$$ {\mathrm{Swelling}}\,{\mathrm{ratio}}\,\left( \% \right) \, = \, \left( {{\mathrm{W}}_{{\mathrm{s}}} - {\mathrm{W}}_{{\mathrm{i}}} } \right)/{\mathrm{W}}_{{\mathrm{i}}} \times { 1}00 $$

where W_s_ represents the weight of the swollen hydrogel, and W_i_ represents its initial weight immediately after in situ gelation. The experiment was conducted in triplicate for each hydrogel type.

To evaluate the ROS scavenging efficacy, superoxide anion (O2^·^) scavenging capability of the hydrogels was assessed using a superoxide dismutase (SOD) assay kit (Dojindo Molecular Technologies, Tokyo, Japan), following the manufacturer's protocol [15]. Briefly, 20 µl of the hydrogel was added to a working solution containing xanthine, xanthine oxidase, and Water-Soluble Tetrazolium (WST)-1, and then incubated at 37 °C for 30 min. The absorbance of WST formazan was measured at 450 nm using a microplate reader, and the SOD activity was calculated as previously described [16]. The ability to generate oxygen was assessed using a dissolved oxygen meter (OxyLiteTM PRO; Oxford Optronix, Adderbury, UK). The prepared hydrogel was immersed in 4 ml of 0.3 and 1 mM H_2_O_2_ solution and incubated at 37 °C for 1 h, after which the dissolved oxygen concentration was measured. The experiment was conducted in triplicate for each hydrogel type.

### Animals

All protocols of animal experiments were approved by the Institutional Animal Care and Use Committee of Seoul National University Hospital Biomedical Research Institute (IACUC no. 24–0050-S1SA1). Animals were maintained in the facility accredited AAALAC International (#001169) in accordance with Guide for the Care and Use of Laboratory Animals 8th edition, NRC (2010).

Twenty male Sprague Dawley Rats, 6 weeks old and weighing 130–190 g, were used for in vivo experiments (a total of 40 ears). The animal was housed in a semi-pathogen-free facility with controlled temperature, humidity, and a light/dark cycle (12 h/12 h). Prior to the experiments, chronic TM perforation was induced [17]. For this, the animal was anesthetized with an intramuscular injection of Zoletil (1.2 ml/kg; Zoletil 50, Virbac, Carros, France) and xylazine (5 mg/kg). After examining the ear under a microscope, a TM perforation was created using a pick involving the inferior quadrant of the TM. Then, mitomycin C-soaked gelatin foam was applied to the perforated wound via the external ear canal for 30 min, with additional applications on days 2 and 4 after TM perforation. Ears with TM perforations that persisted for 5 weeks were selected for further studies using the Ch_MnP hydrogel (Online Resource 1 and 2).

### In vivo hydrogel application

Ears with chronic TM perforation were randomly assigned to three different treatment groups: (1) Control group: ears that received no treatment (n = 20); (2) Ch_MnP(−) group: ears treated with Ch_MnP(−) hydrogel (n = 13); and (3) Ch_MnP(+) group (n = 7): ears treated with Ch_MnP(+) hydrogel. For the groups treated with the hydrogel, we injected 0.03 mL of CS-SH solution and 0.03 mL of the maleimide solution using a dual syringe through a 5 cm mini volume line and a 23-gauge needle via the transtympanic route to reach the bulla, where the hydrogel formed in situ as the mixed solutions exited the needle. The hydrogel administration was performed three times on days 0, 3 and at week 1 for each ear in both the Ch_MnP(−) and Ch_MnP(+) groups.

### Endoscopic TM evaluation

An endoscope (GD-060, Chammed, Gunpo, South Korea), 2.7 mm in diameter, was paired with a smartphone (iPhone 4, Apple Inc., Cupertino, CA, United States) to capture images of the rats’ external auditory canal and TM. Observations were made for signs of inflammation, swelling, congestion, perforation, or other adverse effects. The surface integrity, perforation healing, and transparency of the TM were also evaluated. Perforation size was assessed using Image J (National Institutes of Health, Bethesda, MD, USA). TM endoscopic images were obtained immediately before the hydrogel application (Before hydrogel) and at week 3 after the first hydrogel application.

### Computed tomography (CT) analysis

A micro-CT system (NFR PolarisG90; Nanofocusray, Jeonju, South Korea) was used to assess the remaining hydrogel in the bulla. Images were assessed at the base of the bulla and between the ossicles, where the hydrogel was typically detected. Micro-CT images were processed and evaluated using a DICOM Viewer (Radiant DICOM Viewer Version 2024.1, Poznan, Poland). 2D images were stacked to form a 3D volume. The volume of the remaining hydrogel was calculated by integrating the planimetric measurements of 2D images (mm^2^) and reformatting them into a 3D volume (mm^3^). The micro-CT images were acquired immediately before the hydrogel application (Before hydrogel), immediately after the first hydrogel application (week 0) and week 1 and 3 after the first hydrogel application.

### Auditory brainstem response (ABR) evaluation

The Smart EP system (Intelligent Hearing Systems, Miami, FL, United States) was utilized to measure the ABR threshold at click and 16 kHz frequencies within a soundproof chamber [18, 19]. Before measurements, rats were anesthetized, and subdermal needle electrodes were placed at the vertex (active electrode) and behind both the ipsilateral (reference electrode) and contralateral ears (ground electrode). The speaker was positioned in line with the external auditory canal, and the earphone tube was gently inserted into the ear canal. Hearing thresholds were assessed using click stimuli and 16 kHz pure tone stimuli. The hearing threshold was measured immediately before the hydrogel application (Before hydrogel) and at week 3 after the first hydrogel application, which was compared with the baseline threshold measured from the intact TM before perforation.

### Histology and measurement of the bulla mucosa

All animals were sacrificed at the end point of experiments (week 3) for histological analysis of the bulla mucosa, and external auditory canal (EAC) skin. Bilateral temporal bones were harvested, embedded in paraffin wax, and cut into 5-μm-thick sections. Sections were stained with hematoxylin and eosin. The bulla mucosa and EAC skin were observed with an optical microscope (CX31, Olympus, Tokyo, Japan), and the images were obtained to measure the thickness of bulla mucosa and EAC skin with the DP2-BSW software (Olympus).

### Statistics

Continuous variables were expressed as means ± standard deviations (SD) in the tables and figures. Statistical analyses were performed using SPSS software (version 26.0; SPSS Inc., IBM Corp., Armonk, NY, USA). The Mann–Whitney U test, crosstab and t test were used to compare the outcomes in each group. *p* values < 0.05 were considered to indicate statistical significance.

## Results

### Characterizations of polymers

**Fig. 1:**
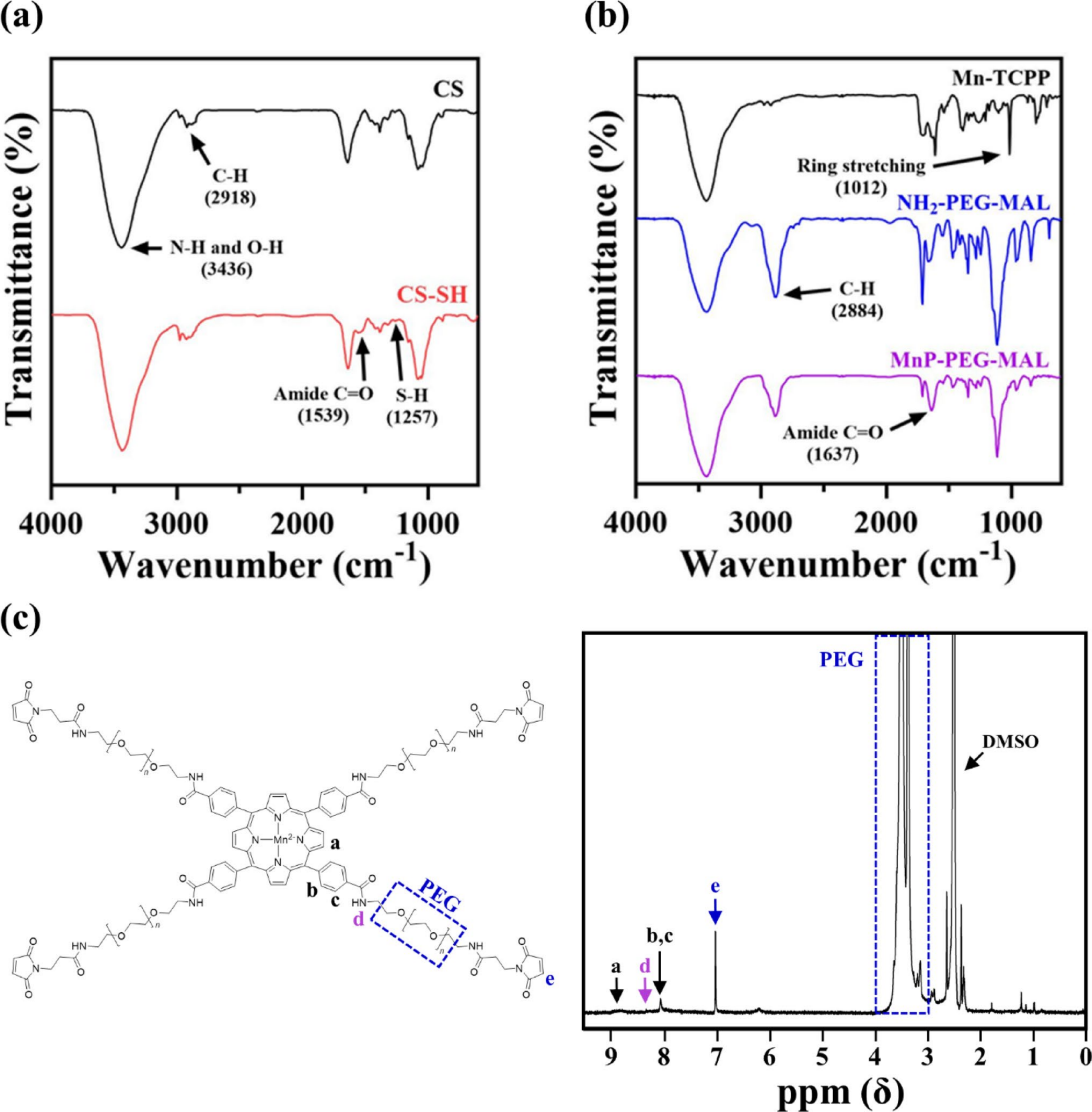
Characterizations of CS-SH and MnP-PEG-MAL. **a** FT-IR spectra of CS and CS-SH. **b** FT-IR spectra of Mn-TCPP, NH_2_-PEG-MAL, and MnP-PEG-MAL. **c** ^1^H NMR spectrum of MnP-PEG-MAL

In order to prepare the in situ hydrogel incorporating MnP, we first synthesized the two distinct functional polymers, CS-SH and MnP-PEG-MAL, employing a synthesis approach adapted from our previous studies with slight modifications [7]. To obtain CS-SH, 3,3-dithiodipropionic acid was first conjugated to CS via amide linkage formation with its amine groups. Subsequent reduction of the disulfide bonds exposed free thiol groups on the polymer backbone. As depicted in Fig. 1a, new characteristic peaks at 1539 and 1257 cm^−1^ in the FT-IR spectrum of CS-SH, corresponding to amide C = O and thiol vibrations, respectively, confirm the successful completion of the two-step modification process [20]. The FT-IR spectrum of native CS displayed characteristic peaks at 3436 cm^−1^, attributed to N − H and O − H stretching [21], and at 2918 cm^−1^, ascribed to C − H stretching [22]. These peaks remained in CS-SH, confirming that the chitosan backbone was retained post-modification. Quantitative thiol analysis using Ellman’s assay determined a thiol content of 562.42 ± 13.02 μmol per gram of polymer, indicating adequate functionality for efficient in situ hydrogel crosslinking via the thiol-maleimide click reaction [23].

MnP-PEG-MAL was obtained through an amide bond formation between the carboxyl groups of Mn-TCPP and the amine groups of NH_2_-PEG-MAL. Figure 1b presents the FT-IR spectrum of MnP-PEG-MAL, showing a new peak at 1637 cm^−1^ associated with C = O stretching, which is indicative of amide bond formation [24]. Additionally, two peaks—one at 1012 cm^−1^ corresponding to the ring stretching vibration of the pyrrole moieties in Mn-TCPP [25], and the other at 2884 cm^−1^ corresponding to the C − H stretching vibrations in NH_2_-PEG-MAL [26]—confirmed the presence of both components in MnP-PEG-MAL. The ^1^H NMR spectrum of MnP-PEG-MAL (Fig. 1c) further validated successful conjugation. Characteristic peaks were observed at 8.8 and 8.0 ppm, attributed to the *β*-pyrrole and phenyl hydrogens of Mn-TCPP, respectively [27]. A peak at 7.0 ppm, assigned to maleimide hydrogens, along with a series of peaks in the 4.0–3.0 ppm region from PEG chain hydrogens, confirmed the incorporation of NH_2_-PEG-MAL [28]. Additionally, a peak at 8.3 ppm corresponded to amide protons formed during the coupling reaction [29].

### Characterizations of hydrogel

**Fig. 2:**
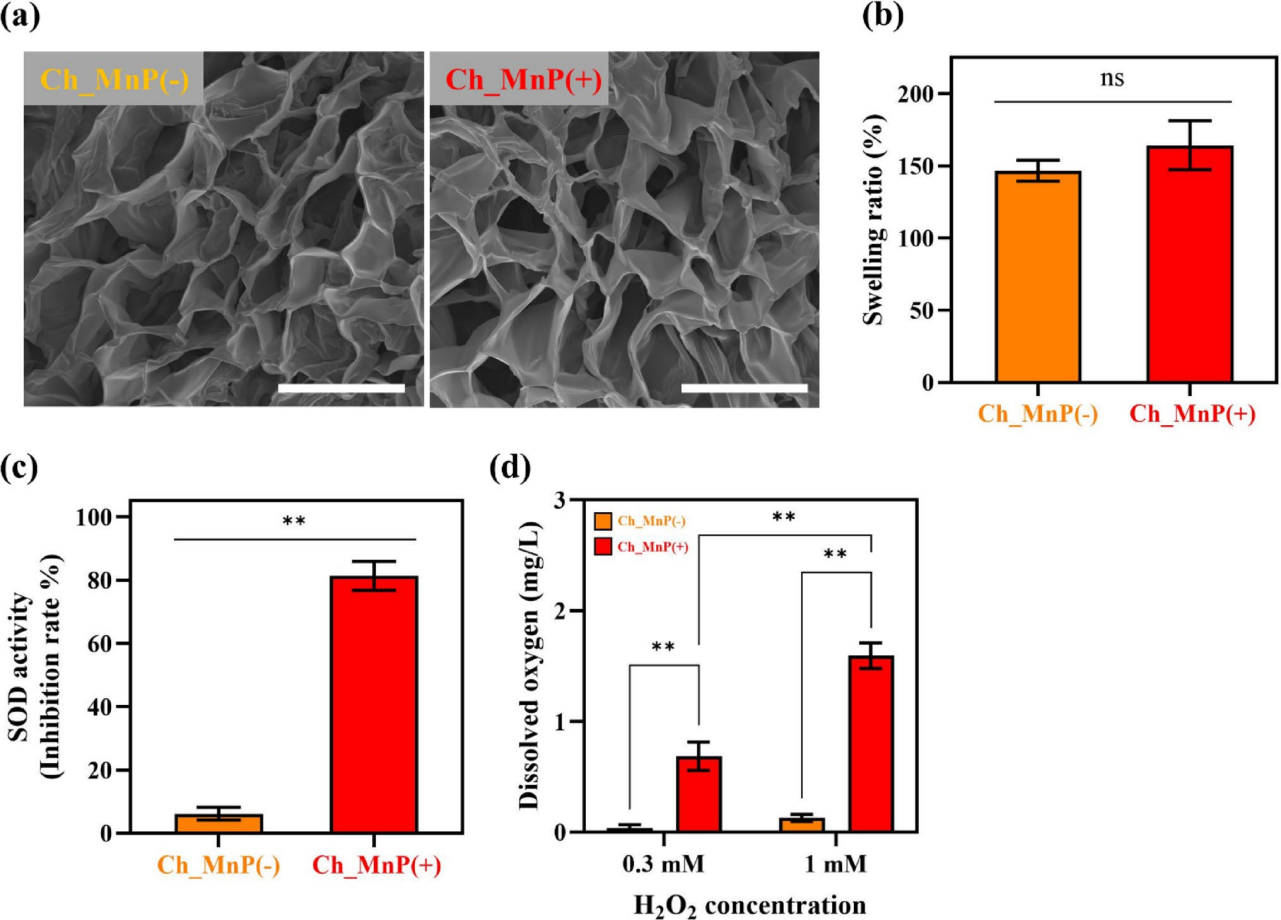
Characterizations of Ch_MnP hydrogels. **a** Representative SEM images. Scale bars are 20 μm. **b** Swelling ratios (*n* = 5). **c** SOD activities (*n* = 5). **d** Dissolved O_2_ concentrations obtained with hydrogels immersed in 0.3 mM and 1 mM H_2_O_2_ solution for 1 h (*n* = 3). Error bars represent the SD. The SOD activities and dissolved O_2_ concentrations of hydrogels were statistically significantly different from each other (*p < 0.05, **p < 0.01)

To prepare the Ch_MnP(+) hydrogel, we mixed the CS-SH solution with a maleimide-containing solution incorporating MnP-PEG-MAL, thereby ensuring the catalytic activity of MnP. In contrast, the Ch_MnP(−) hydrogel, which lacks bioactive MnP, was used as a control. Figure 2a showed that both hydrogels displayed comparable porous structures in their cross sections, leading to similar swelling ratios, as illustrated in Fig. 2b. Both hydrogels absorbed water significantly, reaching a swelling ratio of approximately 155%, which may be optimal for TM wound treatment [30, 31]. Excessive swelling over 500% has been reported to cause hydrogel displacement into the middle ear cavity, potentially leading to hearing loss and impaired healing due to excessive absorption of middle ear discharge [32].

The SOD- and CAT-mimetic activities of the hydrogels were evaluated by measuring their ability to scavenge superoxide anions (O_2_^·–^) and generate O_2_ under ROS-rich conditions mimicking the TM perforation environment [33]. As shown in Fig. 2c, the Ch_MnP(+) hydrogel exhibited significantly enhanced O_2_^·–^ scavenging compared to the MnP-deficient Ch_MnP(−) hydrogel. The Ch_MnP(−) hydrogel showed a slight increase in scavenging capacity, which could be ascribed to minor antioxidant activity inherent to the chitosan matrix itself [34]. Figure 2d demonstrates that O_2_ production was significantly higher in the Ch_MnP(+) hydrogel than in the Ch_MnP(−) hydrogel at H_2_O_2_ concentrations of 0.3 mM and 1 mM, which both fall within the physiological range of ROS levels observed in TM perforations [33]. The Ch_MnP(+) hydrogel significantly produced more dissolved O_2_ at the higher H_2_O_2_ concentration, indicating increased CAT-like activity in response to elevated ROS levels.

### In vivo evaluation

**Fig. 3:**
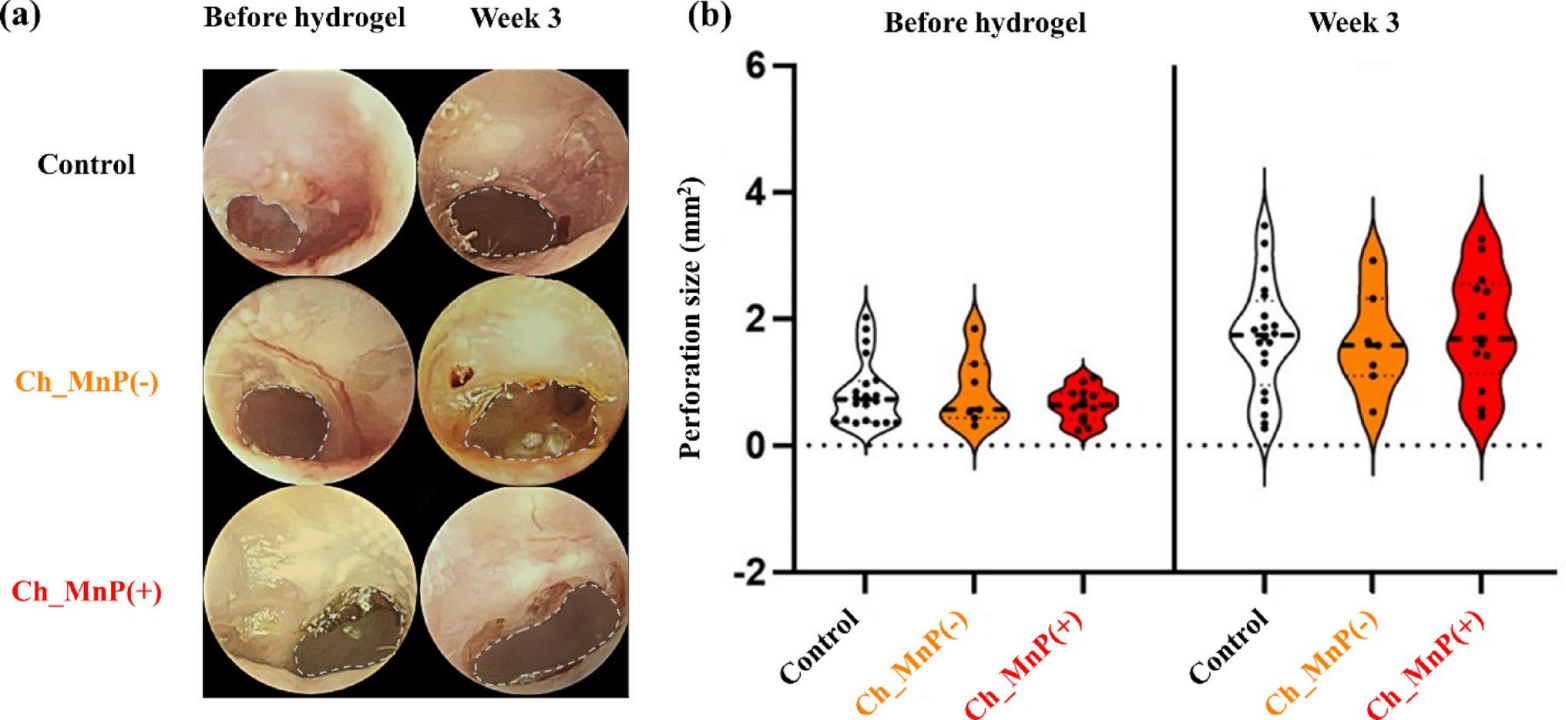
TM perforation size profile by endoscopic image analysis. **a** Representative endoscopic images of the TM with perforation and **b** quantitative analysis of perforation size using violin plots before hydrogel application and at week 3. Perforation areas are outlined with dashed black lines. ns; not statistically significant

To evaluate the efficacy of the proposed hydrogel, we prepared animals with chronic TM perforation and treated them with hydrogel. Figure 3 shows representative endoscopic images and perforation sizes of the TM before hydrogel application and at week 3 after hydrogel application. All animal groups exhibited non-healing TM perforations with similar sizes before hydrogel application. Three weeks after hydrogel application, the size of the TM perforation increased, as observed with the Control group without hydrogel treatment. Before hydrogel application, the size of perforation was 0.26 ± 0.06 mm^2^, 0.29 ± 0.08 mm^2^, and 0.24 ± 0.05 mm^2^ in the Control, Ch_MnP(−), and Ch_MnP(+) groups, respectively, which increased to 0.69 ± 0.55 mm^2^, 0.85 ± 0.55 mm^2^, and 0.64 ± 0.26 mm^2^, respectively. Notably, there were no significant differences in the perforation size among the three groups at week 3 (*p* > 0.05).

**Fig. 4:**
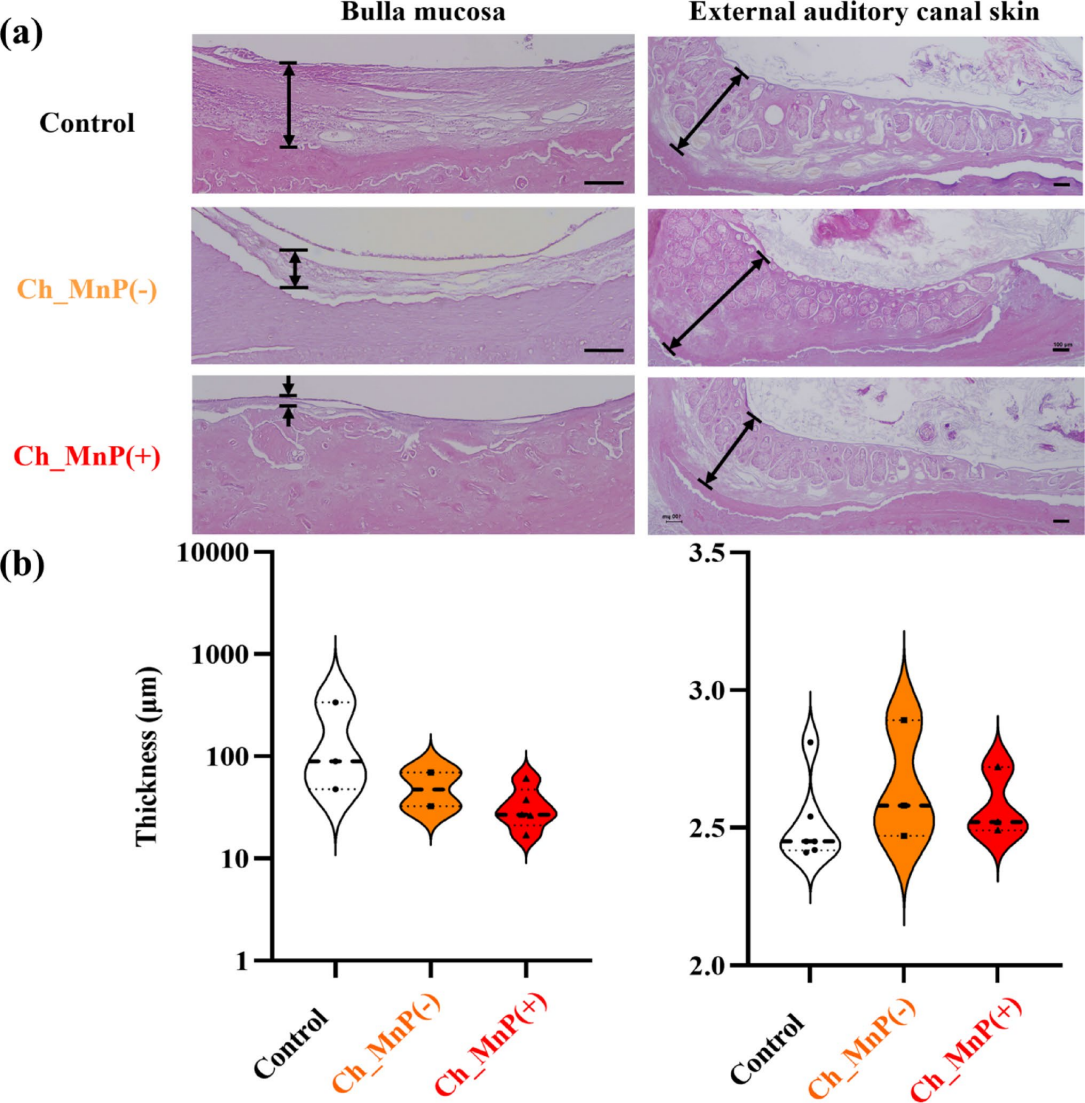
Histological analysis on bulla mucosa and external auditory canal (EAC) skin. **a** Representative H&E-stained tissue images, where the arrows indicate the thickness and **b** their quantitative data. Scale bars are 100 μm. There was no significant difference between all three groups

Accordingly, hearing thresholds did not improve following hydrogel treatment, as shown in Table 1. Before hydrogel application, a significant increase in hearing thresholds was observed across all groups compared to baseline values from the intact TM. By week 3, hearing thresholds in the hydrogel-treated groups remained elevated, with no statistically significant difference compared to the Control group (*p* > 0.05). Histological analysis also showed no statistically significant differences between the hydrogel-treated groups and the Control group in the thickness of the bulla mucosa and EAC skin (Fig. 4). These findings suggest that the hydrogel had no therapeutic efficacy in the TM perforation model used in this study.

**Table 1:** Auditory brainstem response (ABR) hearing threshold

	Click			16 kHz		
	Control	Ch_MnP(−)	Ch_MnP(+)	Control	Ch_MnP(−)	Ch_MnP(+)
Baseline	23.7 ± 2.5	30.0 ± 0.0	25.0 ± 5.0	23.7 ± 2.5	32.5 ± 2.5	22.5 ± 2.5
Before hydrogel	42.5 ± 2.8*	45.0 ± 0.0*	42.5 ± 2.5*	42.5 ± 2.8	40.0 ± 0.0*	45.0 ± 0.0*
Week 3	42.5 ± 2.8*	47.5 ± 2.5*	42.5 ± 2.5*	42.5 ± 2.8	42.5 ± 2.5*	45.0 ± 0.0*

*Significantly different compared to baseline

### Problem analysis

**Fig. 5:**
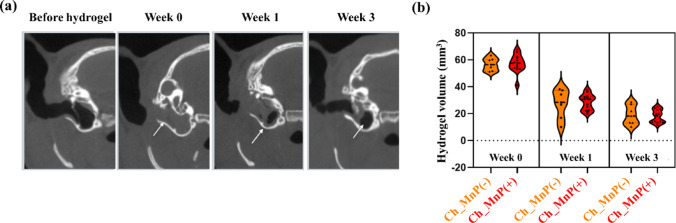
Hydrogel retention profile in the bulla by volumetric CT analysis. **a** Representative micro-CT images showing the middle ear cavity of rats from the Ch_MnP(+) group. **b** Quantitative analysis of residual hydrogel volume over time in both Ch_MnP(−) and Ch_MnP(+) groups. No statistically significant differences were observed between groups

To better understand the lack of therapeutic efficacy, we first assessed hydrogel retention in the ear using micro-CT imaging. As shown in Fig. 5a, both the Ch_MnP(−) and Ch_MnP(+) groups exhibited a well-defined hydrogel filling in the bulla immediately after its application (week 0), with volumes of 56.6 ± 4.6 mm^3^ and 57.3 ± 6.7 mm^3^, respectively. In contrast, images taken before hydrogel application clearly showed an empty bulla, indicating the absence of hydrogel. By week 3, the hydrogel volume visible by CT had dramatically decreased in both groups, measuring 18.9 ± 7.2 mm^3^ in Ch_MnP(−) and 18.1 ± 4.8 mm^3^ in Ch_MnP(+). Given that each ear received three hydrogel applications totaling up to 120 mm^3^ in this experiment, the observed volume reduction of approximately 80% by week 3 suggests substantial water loss from the hydrogel during its residence in the bulla, likely due to exposure to the air-filled environment in the ear [35].

**Fig. 6:**
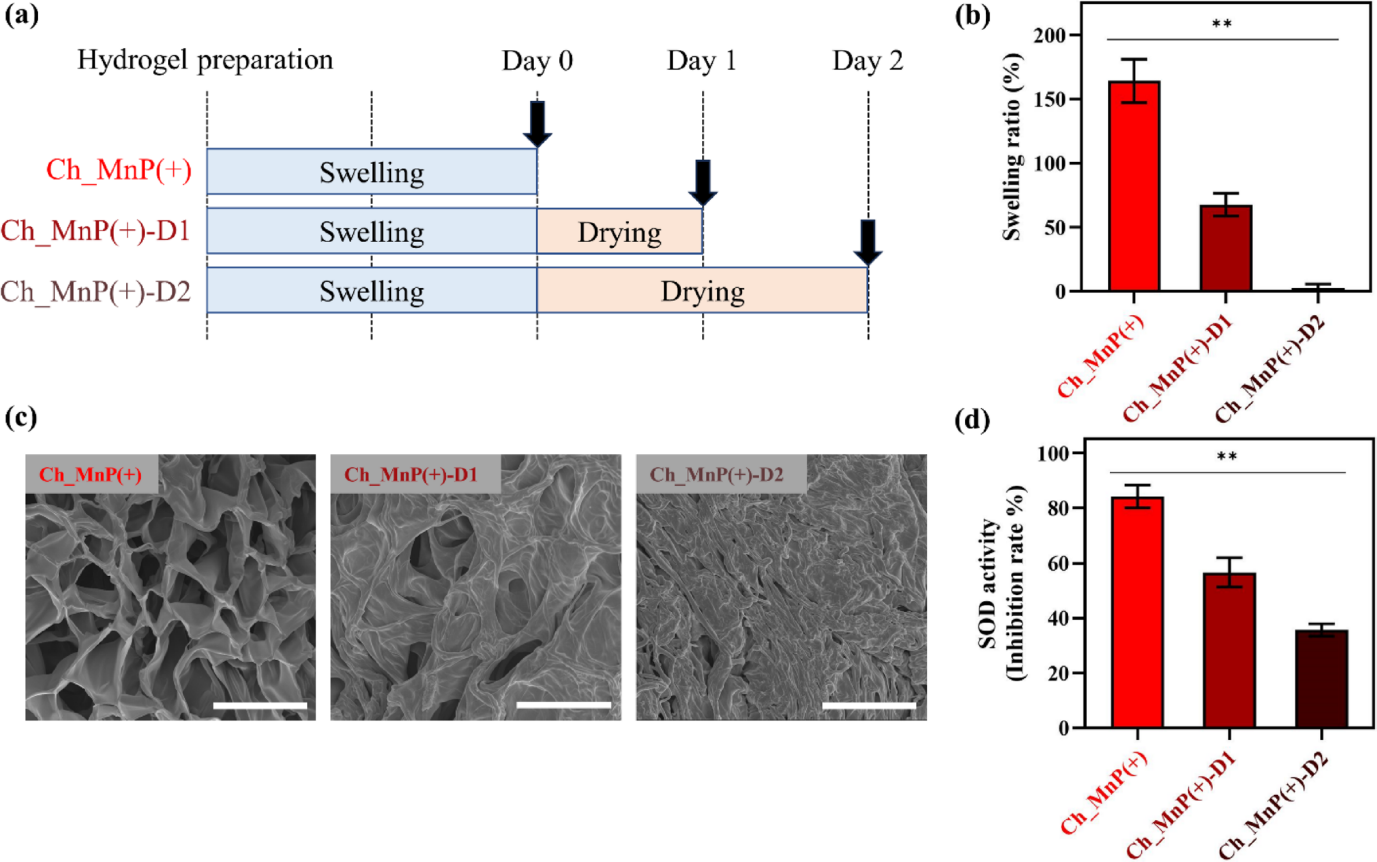
Characterizations of dried functional hydrogels. **a** Dried hydrogel preparation. The Ch_MnP(+) hydrogels were fully swollen in saline for two days, which were dried at room temperature for one and two days to prepare the dried hydrogels of Ch_MnP(+)-D1 and Ch_MnP(+)-D2, respectively. **b** Swelling ratios of dried hydrogels. For this, Ch_MnP(+)-D1 and Ch_MnP(+)-D2 were re-hydrated by immersion in pH 7.4 PBS for two days. **c** Representative SEM images. Scale bars are 20 μm. **d** SOD activities. All error bars represent the SD (n = 5). *Statistically significantly different from each other (**p < 0.01)

Therefore, we examined the characteristics of dried hydrogels, as illustrated in Fig. 6. Our results showed that once the hydrogel lost its water content, it was difficult to rehydrate even in the presence of abundant water (Fig. 6b). This resistance to re-swelling became more pronounced with longer drying durations. As shown in Fig. 6c, dehydration led to shrinkage of the hydrogel network and collapse of the originally present pores (Fig. 2a), with these structural changes becoming increasingly evident with the increase in drying time. Once it collapsed, the network did not return to its original state, thereby hindering rehydration. This structural compromise was accompanied by a decline in functional performance, as indicated by the reduced SOD activity (Fig. 6d). Taken together, the loss of water not only impaired the hydrogel’s structural integrity but also diminished its therapeutic potential.

## Discussion

Many researchers have attempted to develop a non-surgical method to treat chronic TM perforation, but these efforts have been unsuccessful. Ideally, a therapeutic, bioresorbable scaffold that elutes bioagents to accelerate tissue proliferation and healing could be used. Examples of bioagents that have been studied include fibroblast growth factors [36], various subtypes of platelet-derived growth factors [37], hyaluronic acid [38], and epidermal growth factor (EGF) [39]. However, complete closure of perforations has not been achieved with these bioagents alone, and additional surgical procedures were always required [1]. Another important research topic is the route of delivery and the vehicle for loading these bioagents. Although self-administration of liquid form droplets would be convenient for patients, it is likely that the dosage and localization of the drug cannot be customized through self-administration. Transtympanic injection through the perforation seems to be the best delivery route, although an ENT doctor is needed for the procedure [18, 40]. Regarding the vehicle, biocompatible, bioresorbable, and injectable polymers have been studied by our group and others [41, 42]. One interesting study reported that heparin-binding EGF-like growth factor (HB-EGF) loaded in chitosan could regenerate chronic perforations in mouse models with a success rate of 92% [1]. Based on this study, a phase 1/2, randomized, placebo-controlled trial was conducted to assess the safety, tolerability, efficacy, and pharmacokinetics of HB-EGF. Unfortunately, the outcome did not meet the proof of concept for the treatment of chronic TM perforations [43].

Herein, we aimed to address the limitations of previous approaches by developing an in situ hydrogel capable of modulating oxidative stress and hypoxic conditions, both of which are inherently critical factors in chronic TM perforation healing [5, 6]. To achieve this, we designed a chitosan-based hydrogel scaffold immobilized with MnP via click reaction. The hydrogel fabrication followed a protocol similar to our previous studies, with slight modifications to better match the TM tissue environment [7]. CS and MnP were chemically modified to introduce thiol and maleimide groups, respectively, resulting in the formation of CS-SH and MnP-PEG-MAL (Fig. 1). These two precursor solutions were mixed via a Michael Addition click reaction, enabling the in situ formation of the hydrogel. The immobilization of MnP within the biocompatible chitosan hydrogel network enabled the Ch_MnP(+) hydrogel to synergistically scavenge ROS and generate O_2_ in a sustainable manner (Fig. 2c, d). The mechanical properties of the hydrogel, including cross-sectional morphology and swelling ratio, were also designed to be suitable for TM repair (Fig. 2a, b) [32]. Moreover, the cytotoxicity assessment of the hydrogel confirmed its excellent biocompatibility (Online Resource 3), owing to the use of biocompatible materials, including chitosan, Mn-TCPP, and PEG [34, 44]

However, contrary to our expectations, the Ch_MnP(+) hydrogel did not facilitate TM regeneration, despite its previously demonstrated efficacy in bone defect microenvironments [7]. Although the primary goal of this study was not achieved, we identified a key factor that contributed to this outcome: the dry environment of the bulla in the ear, which contrasts with other wound sites characterized by continuous exudate production [45]. The Eustachian tube, which connects the middle ear to the nasopharynx and facilitates middle ear air exchange, may have contributed to constant drying of the hydrogel herein [46]. Because of this, the hydrogel continuously shrank in volume (Fig. 5a), losing contact with the wounded site of the tissue. Once dried, the hydrogel could no longer function effectively as a scaffold, as its pores collapsed and it was unable to rehydrate (Fig. 6b and c) [47]. Furthermore, the loss of water content compromised its SOD catalytic activity, thereby diminishing its therapeutic potential (Fig. 6d). Consequently, no healing effect was observed in TM perforation. These findings underscore the critical importance of maintaining adequate moisture to ensure the optimal performance of the Ch_MnP hydrogel. From a practical standpoint, the hydrogel could, therefore, be supplemented with a humectant, such as glycerol, to enhance water retention [48, 49].

In this study using a small animal model, accurate application of the hydrogel was challenging due to the small size of the rat ear. The high viscosity and limited volume of the hydrogel further complicated precise administration. Ideally, the hydrogel should maintain stable contact with the edge of the TM perforation while allowing keratinocytes to migrate concentrically toward the center. The hydrogel may serve as a scaffold for epithelial cell growth, but only when an appropriate volume is applied and water content is sustainably maintained. Based on our findings, approximately 40–80 μl of hydrogel could be applied to each ear per administration in SD rats. However, controlling this precise amount proved practically difficult. From a clinical perspective, the human middle ear has a volume larger than 400 μl [50], suggesting that hydrogel application may be more feasible and controllable in actual human use. Moreover, this may also allow patients to intermittently apply otic solutions to help prevent water loss during the healing process using the hydrogel [51].

## Conclusion

This study highlights key lessons in developing hydrogel-based therapies for chronic tympanic membrane (TM) perforations. While the Ch_MnP(+) hydrogel showed excellent in vitro performance, it failed to promote TM regeneration in vivo due to dehydration and shrinkage in the dry middle ear environment. Additionally, precise delivery was challenging in the rat ear, leading to suboptimal application. These findings emphasize the need for hydrogels that maintain hydration and structural integrity in dry tissue environments and suggest that the hydrogel may perform better in larger, more accessible human middle ears. Future work should focus on improving hydrogel retention, moisture stability, and delivery methods to maximize its therapeutic potential.

## Supplementary Information

Below is the link to the electronic supplementary material.

Supplementary Material 1
