# Supplementary material for: Challenges in functional hydrogel application for chronic tympanic membrane perforation: practical limitations and lessons learned
Source: Biomed Eng Lett. 2025 Oct 29;16(1):153–65. doi: 10.1007/s13534-025-00519-y (PMC12824086; doi:10.1007/s13534-025-00519-y)
Supplement: Supplementary file 1 — Supplementary Material 1 [file 13534_2025_519_MOESM1_ESM.pdf]

Biomedical Engineering Letters [Supplementary Information]

Challenges in Functional Hydrogel Application for Chronic Tympanic Membrane Perforation: Practical Limitations and Lessons Learned

Gan-Erdene Narantsolmon[1,2,3,†], Yu-Jung Hwang[1,†], Min Ji Kim[4,†], Jin Young Hong[4,5,†], Soo Bin Yoon[4,5], Bayarmaa Enkhbat[3], Chang Hee Min[7], Myoung Ju Kim[4,5], Myung-Whan Suh[1,*], and Young Bin Choy[4,5,6,7,8,9,*]

[1]Department of Otorhinolaryngology, Seoul National University Hospital; Seoul 03080, Republic of Korea

[2]Department of Otorhinolaryngology, First Central Hospital of Mongolia; Ulaanbaatar 210648, Mongolia

[3]Department of Pathology and Forensic Medicine, Mongolian National University of Medical Science; Ulaanbaatar 14210, Mongolia

[4]Interdisciplinary Program in Bioengineering, College of Engineering, Seoul National University; Seoul 08826, Republic of Korea

[5]Integrated Major in Innovative Medical Science, Seoul National University; Seoul 03080, Republic of Korea

[6]Institute of Medical and Biological Engineering, Medical Research Center, Seoul National University; Seoul 03080, Republic of Korea

[7]Department of Biomedical Engineering, Seoul National University College of Medicine; Seoul 03080, Republic of Korea

[8]Innovative Medical Technology Research Institute, Seoul National University Hospital; Seoul 03122, Republic of Korea

[9]ToBIOS Inc., 3F, 9-7 Seongbuk-ro 5-gil, Seongbuk-gu, Seoul 02880, Republic of Korea

[†]These four authors contributed equally as first authors to this work.

Corresponding author Young Bin Choy

E-mail: ybchoy@snu.ac.kr

Corresponding author Myung-Whan Suh

E-mail: drmung@naver.com

This PDF file includes:

Online Resources 1 to 3

Online Resource 1 Auditory brainstem response (ABR) hearing threshold. Week -5 (pre): before tympanic membrane (TM) perforation; Week -5 (post): immediately after TM perforation; Week -3: two weeks after TM perforation. A persistent and marked increase in hearing thresholds was observed following TM perforation, indicating the formation of chronic TM perforation

	**Click**	**16 kHz**
Week -5 (pre)	30.0 ± 0.0	32.5 ± 2.5
Week -5 (post)	35.0 ± 5.0*	40.0 ± 0.0*
Week -3	40.0 ± 0.0*	45.0 ± 0.0*

*Significantly different from Week -5 (pre) ($p < 0.05$)

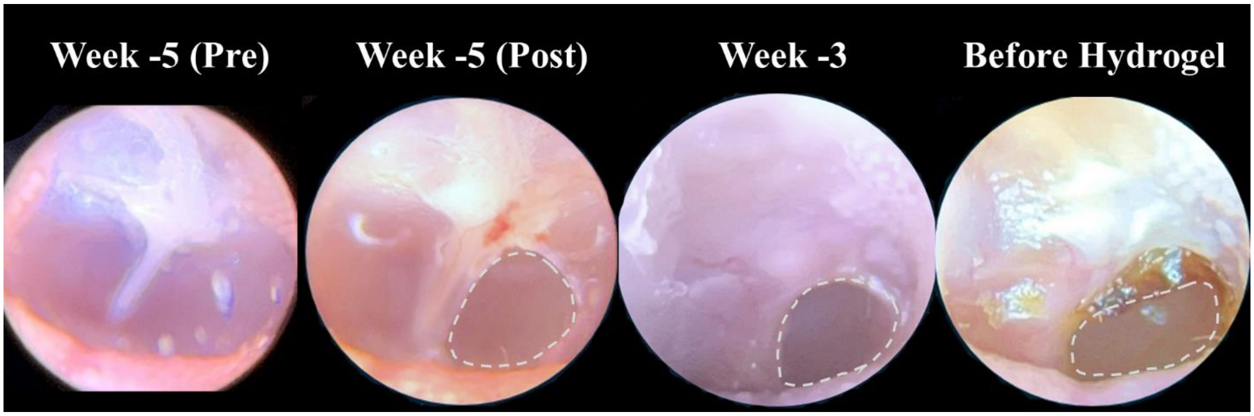

Online Resource 2 Representative otoscopic images of the tympanic membrane (TM) during the induction of chronic TM perforation. Week -5 (pre): before TM perforation; Week -5 (post): immediately after TM perforation; Week -3: two weeks after TM perforation; Before hydrogel: five weeks after TM perforation and immediately before the hydrogel application. The perforated area is outlined with dashed lines. The TM perforation persisted for five weeks without any apparent healing

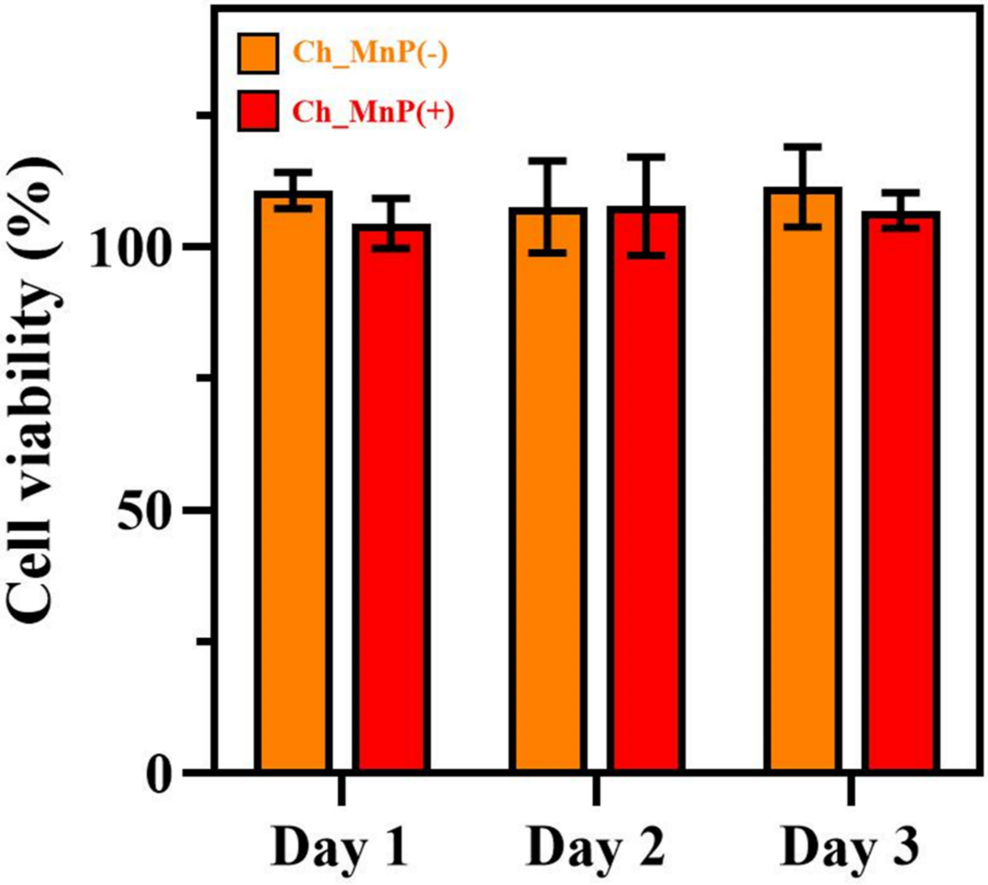

Online Resource 3 *In vitro* cytotoxicity of Ch_MnP(-) and Ch_MnP(+) hydrogels. Error bars represent the SD (n = 6). To assess the biocompatibility of hydrogels, human middle ear epithelial cells (HMEECs) were cultured in a mixture of Dulbecco's modified Eagle's medium (DMEM, Invitrogen, Carlsbad, CA, USA) and bronchial epithelial basal medium (Lonza, Walkersville, MD, USA) (1:1) with 10% fetal bovine serum, 100 units/ml penicillin, and 100 µg/ml streptomycin [1]. The cells were seeded into a 96-well plate at a density of 1×10^4 cells/well and incubated for 24 h at 37 °C in a humidified atmosphere containing 5% CO_2. The hydrogels were sterilized by exposure to ultraviolet light for 30 min, then immersed in 0.3 mL of cell culture medium and incubated at 37 °C under 5% CO_2 for 3 days. At 1, 2, and 3 days, the entire medium was collected and replaced with an equal volume of fresh medium. The collected samples were then analyzed using the EZ-Cytox cell viability assay kit (Daeillab Service, Seoul, Republic of Korea). Briefly, 100 µl of the collected medium was transferred to each well and incubated for 24 h. Afterward, 100 µl of the medium in each well was replaced with fresh medium, and 10 µl of EZ-Cytox reagent was added, followed by a 2-h incubation. Absorbance was measured at 450 nm and 600 nm using a microplate reader (SpectraMax 190 Microplate Reader; Molecular Devices, San Jose, CA). Cell viability was calculated using the following equation [2]:

Cell viability (%) = [(absorbance at 450 nm of the treated well – absorbance at 600 nm of the treated well) / (absorbance at 450 nm of the untreated control well – absorbance at 600 nm of the untreated control well)] × 100 [2]

References

1. Im GJ, Park MK, Song JJ. Effect of urban particles on human middle ear epithelial cells. Int J Pediatr Otorhinolaryngol. 2014;78(5):777-81.
2. Chung CY, Fung SK, Tong KC, Wan PK, Lok CN, Huang Y, et al. A multi-functional PEGylated gold(iii) compound: potent anti-cancer properties and self-assembly into nanostructures for drug co-delivery. Chem Sci. 2017;8(3):1942-53.
